# Draft Genome Sequence of *Aeromonas caviae* CH129, a Marine-Derived Bacterium Isolated from the Coast of São Paulo State, Brazil

**DOI:** 10.1128/genomeA.01336-16

**Published:** 2016-12-01

**Authors:** Flávio Augusto Cardozo, Nadia Catalina Alfonso Vargas, Cristina Kraemer Zimpel, Adalberto Pessoa, Irma Nelly Gutierrez Rivera

**Affiliations:** a Department of Microbiology, Biomedical Sciences Institute, University of São Paulo, São Paulo, São Paulo, Brazil; b Department of Preventive Veterinary Medicine and Animal Health, University of São Paulo, São Paulo, São Paulo, Brazil; c Department of Biochemical and Pharmaceutical Technology, School of Pharmaceutical Sciences, University of São Paulo, São Paulo, São Paulo, Brazil

## Abstract

We report here the draft genome sequence of *Aeromonas caviae* CH129, a marine-derived bacterium isolated from the coast of São Paulo state, Brazil. Genomic analysis revealed genes encoding enzymes involved in binding, transport, and chitin metabolism and different virulence-associated factors.

## GENOME ANNOUNCEMENT

*N*-Acetyl-D-glucosamine (GlcNAc) is a valuable pharmacological agent for the treatment of osteoarthritis and inflammatory bowel disease, including ulcerative colitis and Crohn’s disease (1). This compound has traditionally been produced by the acid hydrolysis of chitin, but the processes are environmentally harmful and have low yield and high cost (2). Therefore, enzymatic production of GlcNAc represents a sustainable alternative to the current processes (3).

We produced GlcNAc from α-chitin using a crude chitinase extract produced by *Aeromonas caviae* CH129, a marine-derived bacterium isolated from the coast of São Paulo state, Brazil. Our results have shown that *A. caviae* CH129 is an efficient chitinase producer strain, but some *A. caviae* strains have been reported to induce pathogenicity in fish and humans (4). Thus, this study aims to sequence and annotate the genome of *A. caviae* CH129, which will serve as a useful genetic reference for the investigation of chitin-degrading enzymes and virulence-associated factors in *A. caviae*.

Genomic DNA from bacterial culture was extracted using a Wizard Genomic DNA purification kit (Promega Corp., Madison, WI, USA) and quantified using a Qubit fluorometer (Life Technologies, Carlsbad, CA, USA). Paired-end and mate-pair libraries were prepared with a Nextera XT DNA library preparation kit (Illumina, Inc., San Diego, CA, USA) and a Nextera mate-pair library preparation kit (Illumina), respectively, and sequenced on a MiSeq platform (Illumina), using the 600-cycle Miseq reagent kit v3 (Illumina). *De novo* genome assembly was performed using the pipeline A5-MiSeq v.20150522 (5) and first-pass annotation was obtained using the NCBI Prokaryotic Genome Annotation Pipeline (PGAP).

A total of 21,199,358 reads were assembled into 26 scaffolds, resulting in a genome size of ~4.42 Mb with a G+C content of 61.6%. Annotation resulted in a total of 3,931 coding sequences (CDSs) and 145 RNAs (32 rRNAs, 107 tRNAs, and six noncoding RNAs [ncRNAs]). Genomic analysis revealed that *A. caviae* CH129 contains genes encoding enzymes involved in binding, transport, and chitin metabolism and different virulence-associated factors, such as hemolysins and RTX toxin. Future comparative analyses will improve our understanding of chitinase diversity and pathogenicity in *A. caviae*.

### Accession number(s).

The draft genome sequence of *A. caviae* CH129 has been deposited at DDBJ/ENA/GenBank under the accession number MDSD00000000. The version described in this paper is the first version, MDSD01000000.
